# A Phylogenomic Study of the Genus Alphavirus Employing Whole Genome Comparison

**DOI:** 10.1002/cfg.478

**Published:** 2005-06

**Authors:** Aimée J. Luers, Sandra D. Adams, John V. Smalley, James J. Campanella

**Affiliations:** 1 Montclair State University Department of Biology and Molecular Biology 1 Normal Avenue Montclair NJ 07043 USA; 2 Bergen Community College Department of Science and Technology 400 Paramus Road Paramus NJ 07652 USA

## Abstract

The phylogenetics of the genus Alphavirus have historically been characterized using partial gene, single gene or partial proteomic data. We have mined cDNA and amino acid sequences from GenBank for all fully sequenced and some partially sequenced alphaviruses and generated phylogenomic analyses of the genus Alphavirus genus, employing capsid encoding structural regions, non-structural coding regions and complete viral genomes. Our studies support the presence of the previously reported recombination event that produced the Western Equine Encephalitis clade, and confirm many of the patterns of geographic radiation and divergence of the multiple species. Our data suggest that the Salmon Pancreatic Disease Virus and Sleeping Disease Virus are sufficiently divergent to form a separate clade from the other alphaviruses. Also, unlike previously reported studies employing limited sequence data for correlation of phylogeny, our results indicate that the Barmah Forest Virus and Middelburg Virus appear to be members of the Semliki Forest clade. Additionally, our analysis indicates that the Southern Elephant Seal Virus is part of the Semliki Forest clade, although still phylogenetically distant from all known members of the genus Alphavirus. Finally, we demonstrate that the whole Rubella viral genome provides an ideal outgroup for phylogenomic studies of the genus Alphavirus.


**Comparative and Functional Genomics**
*Comp Funct Genom* 2005; **6**: 217–227.
Published online in Wiley InterScience (www.interscience.wiley.com). **DOI:** 10.1002/cfg.478

**Research Article**

### A phylogenomic study of the genus Alphavirus employing whole genome comparison

Aimée J. Luers[1], Sandra D. Adams[1], John V. Smalley[2] and James J. Campanella[1]*
[1]*Montclair State University, Department of Biology and Molecular Biology, 1 Normal Avenue, Montclair, NJ 07043, USA*
[2]*Bergen Community College, Department of Science and Technology, 400 Paramus Road, Paramus, NJ 07652, USA*

**Correspondence to: James J. Campanella, Montclair State University, Department of Biology and Molecular Biology, 1 Normal Avenue, Montclair, NJ 07043, USA. E-mail: james.campanella@montclair.edu*

Received: 30 June 2004
Revised: 6 January 2005
Accepted: 17 March 2005

### Abstract

**The phylogenetics of the genus Alphavirus have historically been characterized using partial gene, single gene or partial proteomic data. We have mined cDNA and amino acid sequences from GenBank for all fully sequenced and some partially sequenced alphaviruses and generated phylogenomic analyses of the genus Alphavirus genus, employing capsid encoding structural regions, non-structural coding regions and complete viral genomes. Our studies support the presence of the previously reported recombination event that produced the Western Equine Encephalitis clade, and confirm many of the patterns of geographic radiation and divergence of the multiple species. Our data suggest that the Salmon Pancreatic Disease Virus and Sleeping Disease Virus are sufficiently divergent to form a separate clade from the other alphaviruses. Also, unlike previously reported studies employing limited sequence data for correlation of phylogeny, our results indicate that the Barmah Forest Virus and Middelburg Virus appear to be members of the Semliki Forest clade. Additionally, our analysis indicates that the Southern Elephant Seal Virus is part of the Semliki Forest clade, although still phylogenetically distant from all known members of the genus Alphavirus. Finally, we demonstrate that the whole Rubella viral genome provides an ideal outgroup for phylogenomic studies of the genus Alphavirus. Copyright © 2005 John Wiley & Sons, Ltd.**

**Keywords:** **phylogenetics; phylogenomics; Alphavirus; Togaviridae; Sindbis Virus; Semliki Forest Virus**

**Supplementary material for this article can be found at: http://www.interscience.wiley.com/jpages/1531-6912/suppmat/**

### Introduction

Alphaviruses comprise a genus of arboviruses of the Family *Togaviridae* that infect many different vertebrate hosts and are transmitted by a number of invertebrate vectors. Nearly 30 different alphaviruses have been isolated worldwide and classified into one of seven serocomplexes (Calisher and Karabatsos, 1988; Powers *et al.*, 2001). These species cause disease in a wide range of animals and humans. Based on the geographic location in which the specific alphaviruses have been isolated, each species has been described as Old World (Asia, Australia, Europe, and Africa) or New World (North America and South America) (Strauss and Strauss, 1994). The Alphavirus genome is a positive-strand RNA molecule, approximately 11–12 kb in length. The 5′ end of the genome encodes the non-structural proteins nsP1–nsP4. The 3′ terminal region encodes the structural proteins: capsid, 6K and envelope genes 1–3 (Strauss and Strauss, 1994).

Large-scale genomic recombination events between an Eastern Equine Encephalitis Virus (EEEV) and a Sindbis-like ancestor are hypothesized to

Copyright © 2005 John Wiley & Sons, Ltd.

**Table 1.** Viral species employed in this study

| Complex and viruses | Abbreviation | GenBank Accession Nos | | |
|---|---|---|---|---|
| | | Structural region | Non-structural region | Complete genome |
| Barmah Forest complex | | | | |
| Barmah Forest Virus | BFV | 28 193 962 | 9 629 246 | 9 629 246 |
| Eastern Equine Encephalitis complex | | | | |
| Eastern Equine Encephalitis Virus | EEEV (NA)* | 21 218 484 | 21 218 484 | 21 218 484 |
| | (SA) | 6 166 360 | | |
| Middelburg Virus complex | | | | |
| Middelburg Virus | MIDV | 28 193 965 | | |
| Semliki Forest complex | | | | |
| Chikungunya Virus | CHIKV | 28 193 962 | 27 754 751 | 27 754 751 |
| Mayaro Virus | MAYV | 19 073 904 | 19 073 904 | 19 073 904 |
| O'Nyong Nyong Virus | ONN | 9 627 007 | 9 627 007 | 9 627 007 |
| Subtype: Igbo-Ora Virus | IOV | 9 630 653 | 9 630 653 | 963 065 |
| Ross River Virus | RRV | 333 914 | 9 790 297 | 9 790 297 |
| Subtype: Sagiyama Virus | SAGV | 28 193 956 | 7 288 147 | 7 288 147 |
| Semliki Forest Virus | SFV | 16 767 845 | 16 767 845 | 16 767 845 |
| Venezuelan Equine Encephalitis complex | | | | |
| Venezuelan Equine Encephalitis Virus | VEEV | 5 001 414 | 9 626 526 | 9 626 526 |
| Western Equine Encephalitis complex | | | | |
| Aura Virus | AURA | 21 218 488 | 21 218 488 | 21 218 488 |
| Sindbis Virus | SIN | 20 086 759 | 9 790 313 | 9 790 313 |
| Sindbis (Ockelbo Virus) | OCKV | 334 111 | 334 111 | 334 111 |
| Recombinants | | | | |
| Buggy Creek Virus | BCV | 28 193 929 | | |
| Fort Morgan Virus | FMV | 28 193 932 | | |
| Highlands J Virus | HJ | 28 193 935 | | |
| Western Equine Encephalitis Virus | WEEV | 21 238 454 | 21 238 454 | 21 238 454 |
| 'Unclassified' alphaviruses | | | | |
| Salmon Pancreatic Disease Virus | SPDV | 4 808 418 | 21 321 727 | 21 321 727 |
| Sleeping Disease Virus | SDV | 6 138 913 | 19 352 423 | 19 352 423 |
| Southern Elephant Seal Virus | SESV | 12 964 698 | | |

* NA, North America; SA, South America.

have resulted in the production of a 'Western Equine Encephalitis Virus' (WEEV) (Hahn *et al.*, 1988; Levinson *et al.*, 1990; Weaver *et al.*, 1993; Strauss and Strauss, 1997) (Table 1). In turn, this virus speciated into a number of discrete alphaviruses, including the Highlands J, Buggy Creek, and Fort Morgan Viruses (Calisher *et al.*, 1980, 1988; Strauss and Strauss, 1997). These alphaviruses, along with Sindbis, have been classified into the 'WEE complex', based upon serologic analyses (Calisher *et al.*, 1980, 1988) and confirmed by sequence alignments (Hahn *et al.*, 1988) and limited phylogenetic analysis (Levinson *et al.*, 1990; Weaver *et al.*, 1997; Powers *et al.*, 2001). The WEEV descendants contain the E1 and E2 structural proteins from the Sindbis-like progenitor; the remainder of the genome retains EEEV sequence similarities.

Current alphaviral study includes molecular characterization of the genes, development of alphaviral-based vectors and vaccines, and phylogenetic characterization of the lineage of the alphaviruses (Strauss and Strauss, 1994; Frolov *et al.*, 1996; Schlesinger and Dubensky, 1999; Powers *et al.*, 2001). Recent advances in automated sequencing have produced a plethora of sequence information that allows review and revision of the earlier immunological results that have categorized alphaviruses into the seven antigenic complexes (Calisher and Karabatsos, 1988).

Calisher *et al.* (1980) identified Western Equine Encephalitis complex viruses, which led to an antigenic classification of the WEE complex viruses that has remained unaltered (Calisher and Karabatsos, 1988; Strauss and Strauss, 1994; Powers *et al.*, 2001). A subsequent re-evaluation of

Copyright © 2005 John Wiley & Sons, Ltd. 

neutralization testing (Calisher *et al*., 1988) further distinguished what would come to be known as 'WEE complex recombinants' from other complex members, including Sindbis, its subtypes, and Aura (Powers *et al*., 2001).

The first phylogenetic analyses comparing the regions of recombination of Sindbis and Eastern Equine Encephalitis Viruses were performed by Hahn *et al*. (1988). The study compared amino acid identity of the nsP4 carboxyterminus, capsid, E1, E2, E3 and 6K proteins of the EEEV, SIN, Western Equine Encephalitis and Venezuelan Equine Encephalitis viruses.

Analysing non-structural protein differences, Weaver *et al*. (1993) found the presence of two major alphaviral groups: the Old World viruses (Sindbis, O'Nyong Nyong, Middelburg, Ross River, and Semliki Forest viruses) and the New World viruses (EEEV, VEEV, WEEV). Later, Weaver *et al*. (1997) determined alphaviral phylogenetic relationships for the WEE complex based on 500 nt-length portions of the C-terminal regions of both the E1 and nsP4 genes.

Salmon Pancreatic Disease Virus (SPDV) and Sleeping Disease Virus (SDV) are two of the more recently discovered alphaviruses (Boucher *et al*., 1994; Boucher and Laurencin, 1996; Christie *et al*., 1998). Antigenic similarity of these species was determined through immune cross-protection (Boucher and Laurencin, 1996), neutralization and histopathological testing (Weston *et al*., 2002). Limited phylogenetic analysis was performed on these species (Villoing *et al*. 2000; Weston *et al*. 2002). Another new virus, the poorly understood Southern Elephant Seal Virus (SESV), was phylogenetically compared to the viruses of the Semliki Forest (SF) complex and shown to antigenically cross-react with other members from this geographic region.

Powers *et al*. (2001) produced a comprehensive phylogram that included almost all known alphaviral strains and many subtypes. This phylogram was generated on the basis of a portion of the E1 glycoprotein sequence and grouped the alphaviruses into their antigenic complexes. The phylogram also indicated points at which the viruses may have been geographically translocated between the Old and New Worlds.

Previous phylogenetic studies have been limited by the methodologies and sequences employed. For example, the antigenic complexes formulated (Calisher and Karabatsos, 1988) are based upon E1, E2, and capsid glycoprotein immunological relationships between the viruses, but ignore the rest of the genome. The partial gene sequences used to produce identity and cladistic data do not reflect whole viral genome similarity (Hahn *et al*., 1988; Levinson *et al*., 1990; Weaver *et al*., 1993, 1994, 1997; Powers *et al*., 2001). Therefore, the antigenic complexes used to categorize the relationships within the genus Alphavirus are restricted in their ability to fully identify phylogenomic relationships.

Building upon the work of Levinson *et al*. (1990), we deduced phylogenomic relationships between all sequenced alphaviruses, using complete non-structural, structural and whole genomic cDNA and amino acid data. These data were used to generate phylograms that could be compared to the published phylogenetic trees of Levinson *et al*. (1990), Weaver *et al*. (1993, 1997) and Powers *et al*. (2001). Additionally, we have proposed the appropriate phylogenetic positions of the three newly recognized alphaviruses (SPDV, SDV and SESV).

### Materials and methods

### Sources of sequence data

All Alphavirus and Rubella virus (Accession No. 9 790 308) sequences were obtained from the National Center for Biotechnology Information (NCBI) GenBank database (**www.ncbi.nlm.nih.gov**), using the 'Nucleotide' search option (Table 1). The sequences were converted to FASTA format using the 'FASTA' display option and saved as individual text files. All sequences were combined into a single text file for alignment procedures. This process was repeated for the structural polyprotein cDNA sequences of the alphaviruses of interest (Table 1).

The cDNA sequences of the non-structural polyprotein coding region were identified by aligning the nsP4 cDNA region of the Alphavirus in question to the complete genome. Alignments were performed using the BLAST pairwise alignment (**www.ncbi.nlm.nih.gov/blast/bl2seq/bl2.html**). Alignment analysis revealed the appropriate nucleotide at which the non-structural region ended for each species. The promoter and structural sequences were removed from the overall sequence

Copyright © 2005 John Wiley & Sons, Ltd. 

by hand and the derived non-structural regions combined into a single FASTA file.

Amino acid viral sequences were obtained from NCBI GenBank database (**www.ncbi.nlm.nih.gov**) (Table 1). The structural and non-structural polyprotein amino acid sequences were available for both the alphaviruses and Rubella virus. These were copied and pasted into a FASTA file. The structural and non-structural protein sequences, after data mining, were divided into separate files. For each species, full-length genomic amino acid data was produced by hand, joining the amino terminal of the non-structural polyprotein sequence to the carboxyterminal amino acid of the structural polyprotein sequence. These complete amino acid sequences were combined into a FASTA file.

### Sequence alignments and phylogenetic tree construction

Multiple alignments of all DNA and amino acid sequences were constructed using the Clustal X v1.81 software (Thompson *et al.*, 1997). All alignments were performed using the default values of the Clustal X program. The DNA and protein sequences of Rubella virus were employed as outgroups in all studies.

Phylogenetic trees were generated from the distances provided by the Clustal X analysis using the neighbour-joining method (Saitou and Nei, 1987). Bootstrap analyses (Felsenstein, 1985) consisted of 1000 replicates. The neighbour-joining trees were visualized with the TREEVIEW program (Page, 1996). All bootstrap values of less than 500 are not shown on phylograms.

### Pairwise alignment

The Matrix Global Alignment Tool (MatGAT) v. 2.01 was used to compare the viral cDNA and protein sequences in pairwise analyses (Campanella *et al.*, 2003). All cDNA sequences were evaluated using a first gap penalty of '70' and an extending gap penalty of '1', while all protein sequences were compared using the program defaults.

### GenDistance analysis

The complete genomic sequences for each Alphavirus and Rubella virus were converted to FASTA files and analysed using the default settings of GenDistance (Chen *et al.*, 2000). The output file was bootstrapped using the neighbour-joining function of the PHYLIP package (Felsenstein, 1985) and visualized as a rectangular cladogram using TreeView.

### Results

### Comparison of complete genomic cDNA and amino acid sequences

The complete genomic phylograms reveal three specific clades: the Semliki Forest (SF) clade, the Sindbis–Equine Encephalitis (SEE) clade, and the Aquatic Virus (AV) clade (Figure 1). The shorter branch lengths of the amino acid phylogram (Figure 1A) compared to those of the genomic cDNA phylogram (Figure 1B) suggest degenerative conservation of the amino acid sequences. Barmah Forest Virus groups with the SEE clade in the amino acid phylogram (bootstrap value 1000) and further, Igbo-Ora and O'Nyong Nyong Viruses diverge from Chikungunya Virus (Figure 1).

Rubella virus acts as a distant, but viable, outgroup because it stems from a separate node in the phylograms and its genes share similar functions to those of the genus Alphavirus (Frey, 1994) (Figure 1). Additionally, the identity matrix analysis reveals that Rubella virus is equally genetically distant from all alphaviruses. The genomic identity values for Rubella virus averaged 28.7%, while the amino acid values averaged 17.9% (on-line supplementary materials, Figure A), supporting the use of the Rubella virus as a practical outgroup in phylogenomic studies.

The members of the AV clade also demonstrate an 'outgroup-like' distance from the other members of the genus Alphavirus (Figure 1). Compared to the other alphaviruses, the Aquatic viruses (Salmon Pancreatic Disease Virus and Sleeping Disease Virus) demonstrate average cDNA and protein identity values of 43.8% and 38.5%, respectively (on-line supplementary materials, Figure A).

There are two subclades with values that indicate close genetic distance between clade members. All members of the Sindbis subclade of the SEE clade show an average cDNA identity of 72.2% and amino acid identity of 76.5% (on-line supplementary materials, Figure A). Individual protein identity values for the SEE clade are all greater than 50%, suggesting sufficient genetic distance for member inclusion in this clade. The members of

Copyright © 2005 John Wiley & Sons, Ltd. 

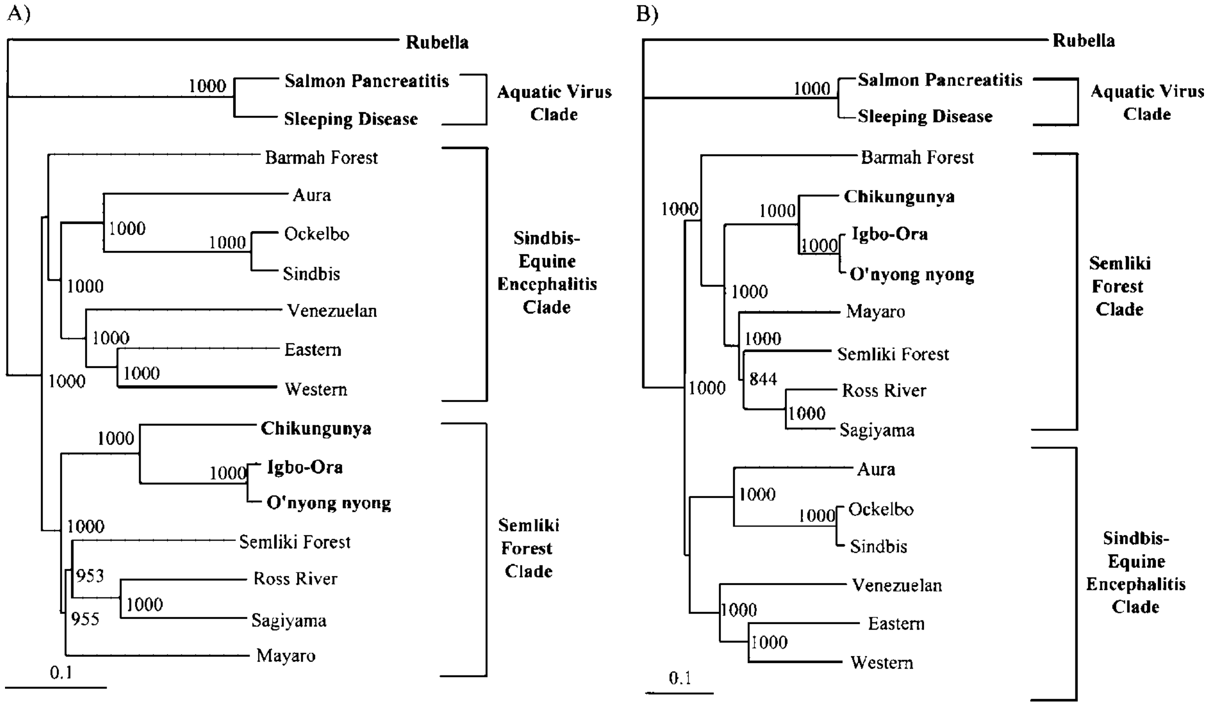

**Figure 1.** Phylograms examining the complete genomes of the alphaviruses. These trees were generated using Clustal X and neighbour-joining analysis (Thompson *et al.*, 1997; Saitou and Nei, 1987). Each of the alphaviral clades are identified in bold. Rubella, Salmon Pancreatitis, and Sleeping Disease Viruses are in bold to denote their placement as outgroups. A) Whole cDNA alphaviral genomic sequences. B) Whole amino acid alphaviral genomic sequences the SF clade have cDNA identity values ranging from 53% to 97% with an average value of 63% (on-line supplementary materials, Figure A, upper matrix). The average protein identity value for the SF clade members is 65.5% (on-line supplementary materials, Figure A, lower matrix).

As an alternative method of cDNA sequence analysis, the GenDistance program (Chen *et al.*, 2000) was employed to compare the genomes of viral species. GenDistance utilizes data compression as a tool to retrieve information in genetic sequences. Genetic distance values are determined using shared information between compressed data sequences and calculated relatedness. The GenDistance generated data was used to produce a phylogram that exhibits the same three clades (SF, Sindbis–Eastern Encephalitis Virus and Aquatic Virus) as were found using the sequence alignment assay of Clustal X (on-line supplementary materials, Figure B). This result provides independent support that our standard alignment analysis has generated phylogenies that are reliable and credible. Additionally, Rubella virus again acts as an appropriate outgroup in the GenDistance cladogram. Barmah Forest Virus is located within the SF clade of the GenDistance cladogram, confirming its membership in the SF clade. Finally, Igbo-Ora Virus and O'Nyong Nyong Virus diverge from Chikungunya.

### Comparison of alphaviral structural protein sequences

The structural phylograms comparing cDNA and amino acid sequences indicate the same major three clades seen previously (Figure 2). Both phylograms (Figure 2) show the same divergence into the SF and SEE clades, with a bootstrap value of 1000. The SF clade contains similar groupings as the genomic phylograms, with the exceptions of O'Nyong Nyong Virus, diverging before Chikungunya Virus and Igbo-Ora Virus. Note that the Southern Elephant Seal, Barmah Forest and Middelburg Virus species are included in the SF clade.

The SEE clade is characterized by three smaller groupings in both amino acid and cDNA phylograms: the Sindbis, 'Recombinant', and the

Copyright © 2005 John Wiley & Sons, Ltd.

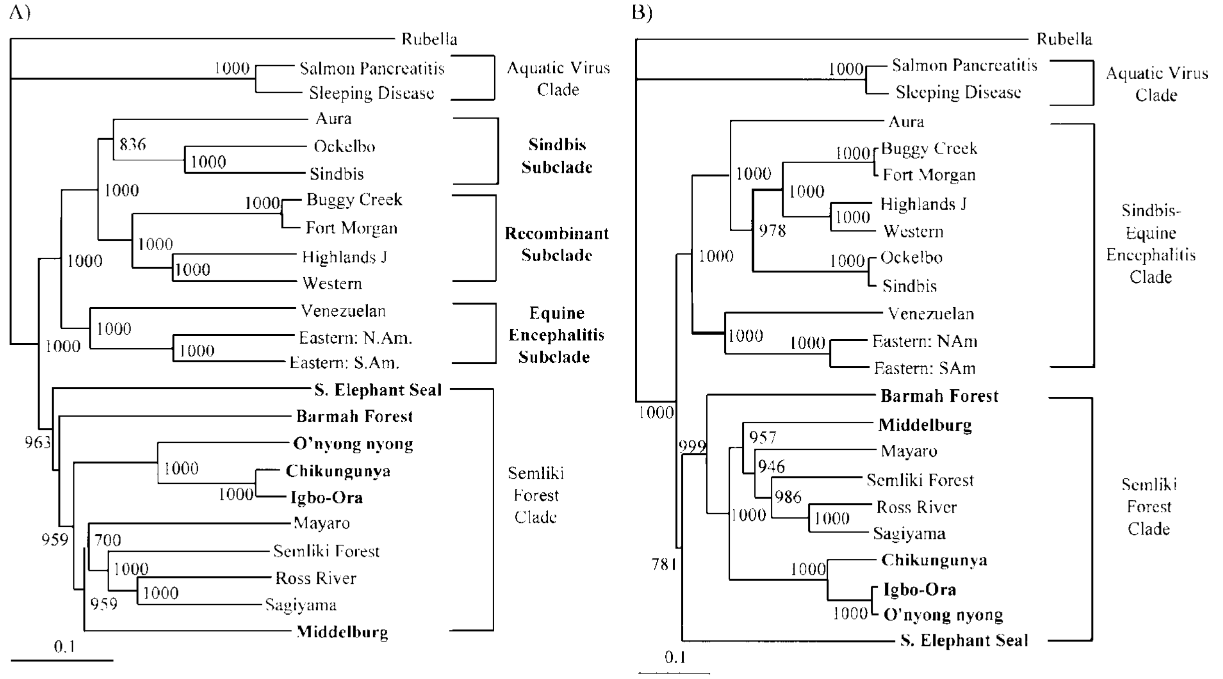

**Figure 2.** Phylograms of alphaviral structural polyproteins generated using neighbour-joining analysis. The subclades of the Sindbis-Equine Encephalitis Clade are denoted in bold. The placement of Southern Elephant Seal, Barmah Forest, and Middelburg Viruses are noted. A) cDNA sequences. Chikungunya and Igbo-Ora Viruses diverge from O'nyong nyong Virus. B) Amino acid sequences. Igbo-Ora and O'nyong nyong Viruses diverge from Chikungunya Virus

Equine Encephalitis subclades (Figure 2). The branch where the SEE clade diverges from the SF clade has a bootstrap value of 1000, which is consistent with the results of the genomic phylogram (Figure 1).

The identity percentages for all members of the SF clade are greater than 50% with the amino acid matrix showing similar values (on-line supplementary materials, Figure C, lower matrix). Barmah Forest Virus has the closest cDNA identity percentages to Middelburg, Ross River and Sagiyama Viruses (on-line supplementary materials, Figure C, upper matrix); the amino acid identity percentages are similar to the aforementioned viruses as well as to Chikungunya, Igbo-Ora, O'Nyong Nyong, Mayaro and SF viruses. Middelburg Virus demonstrates the highest identity with BFV, MAYV, RRV, SAGV and SFV. The members of the Sindbis, Equine Encephalitis, and Recombinant subclades show highest identity values when compared to each other, e.g. Sindbis and Ockelbo viruses.

When compared to the other alphaviruses, the cDNA identities of Southern Elephant Seal Virus (SESV) have an average of 22.8%; the average identity of the SESV amino acid alignments is 20.9% (on-line supplementary materials, Figure C). In light of the low identity values that SESV demonstrates against every Alphavirus examined, it became necessary to conclusively determine its phylogenomic placement by aligning the structural polyprotein sequences of all potential members of the SF clade. SESV appears significantly distant from all other members of this clade (Figure 3). Further, the placement of Barmah Forest and Middelburg viruses into the SF clade are confirmed by the bootstrap values (all over 900) of this phylogram (Figure 3).

### Comparison of alphaviral non-structural protein sequences

Comparisons of the non-structural polyproteins demonstrate somewhat different results from the genomic analysis (Figure 4). The Equine Encephalitis subclade, Sindbis subclade and the Semliki Forest Clade are maintained while the Recombinant Clade is no longer in evidence. Further, the EE

Copyright © 2005 John Wiley & Sons, Ltd. 

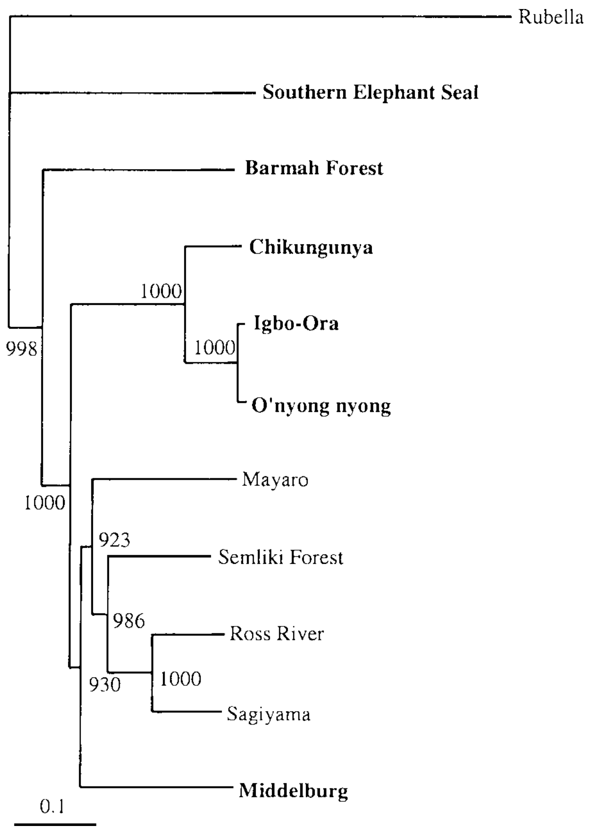

**Figure 3.** A phylogram of Semliki Forest Clade structural polyprotein amino acid sequences, demonstrating that Southern Elephant Seal Virus is phylogenomically distant from the other members of the clade. This tree was generated employing neighbour-joining analysis. The positions of Southern Elephant Seal, Barmah Forest, Chikungunya, Igbo-Ora, O'nyong nyong, and Middelburg Viruses are identified in bold subclade diverges prior to the SIN subclade and the SF Clade. Like the complete genomic phylogram (Figure 1), Igbo-Ora and O'nyong nyong Viruses diverge from Chikungunya Virus (Figure 4).

The cDNA identity values are comparable for all the alphaviruses to which Barmah Forest Virus is compared (on-line supplementary materials, Figure D). Western Equine Encephalitis Virus is closer to Venezuelan Equine Encephalitis Virus and Eastern Equine Encephalitis Virus than any other Alphavirus compared. The amino acid matrix results reveal highest identity values when BFV is compared to Igbo-Ora Virus (on-line supplementary materials, Figure D). WEEV demonstrates the closest cDNA identity values to VEEV and EEEV, while it also shares amino acid identity with most of the alphaviruses compared. This supports the Hahn *et al.* (1988) assertion that the non-structural polyprotein region of the WEEV genome retains homology with its EEEV progenitor.

### Discussion

The antigenic complexes currently used within the alphaviral literature do not elucidate the extent to which these species are related genetically, nor do they provide a taxonomical designation for the most recently isolated alphaviruses. Though the studies of Powers *et al.* (2001) incorporated portions of genes, it is the most comprehensive alphaviral phylogenetic project to date. This work, however, did not appropriately assign the viruses into descriptive clades, nor did it use a sufficiently divergent virus as an outgroup to root phylogenetic analyses. In the present study, we have addressed these issues and recommended changes to alphaviral phylogenetics. We have revised the phylogenetic relationships of the Western Equine Encephalitis Complex, Barmah Forest, Middelburg, Salmon Pancreatic Disease, Sleeping Disease, and Southern Elephant Seal Viruses (Table 2). The complete genomic cDNA and amino acid analyses described represent the first time that whole genomic sequences have been utilized for phylogenomic analysis of alphaviruses. Our analyses address how to describe overall genetic relatedness of a genus of viruses that utilize myriad hosts and vectors within their various replication cycles and have speciated through recombination, divergence, and radiation. Complete genomic analysis widens the methodological focus with which alphaviruses are presently examined.

Our 'complete genomic' phylograms separate the alphaviruses studied into three major phylogenomic clades: the Semliki Forest (SF), the Sindbis-Equine Encephalitis (SEE), and the Aquatic Virus (AV) Clades (Figure 1). These phylograms allow conclusive classification of Salmon Pancreatic Disease and Sleeping Disease Viruses into their own clade. These results are internally consistent throughout our studies (Figs. 1, 2, and 4; on-line supplementary materials Figs. A-D), supporting our conclusions. The classification of SPDV and SDV into their own clade is underscored by the unique pathophysiological manifestations of these diseases (Boucher and Laurencin, 1996). Therefore, the

Copyright © 2005 John Wiley & Sons, Ltd.

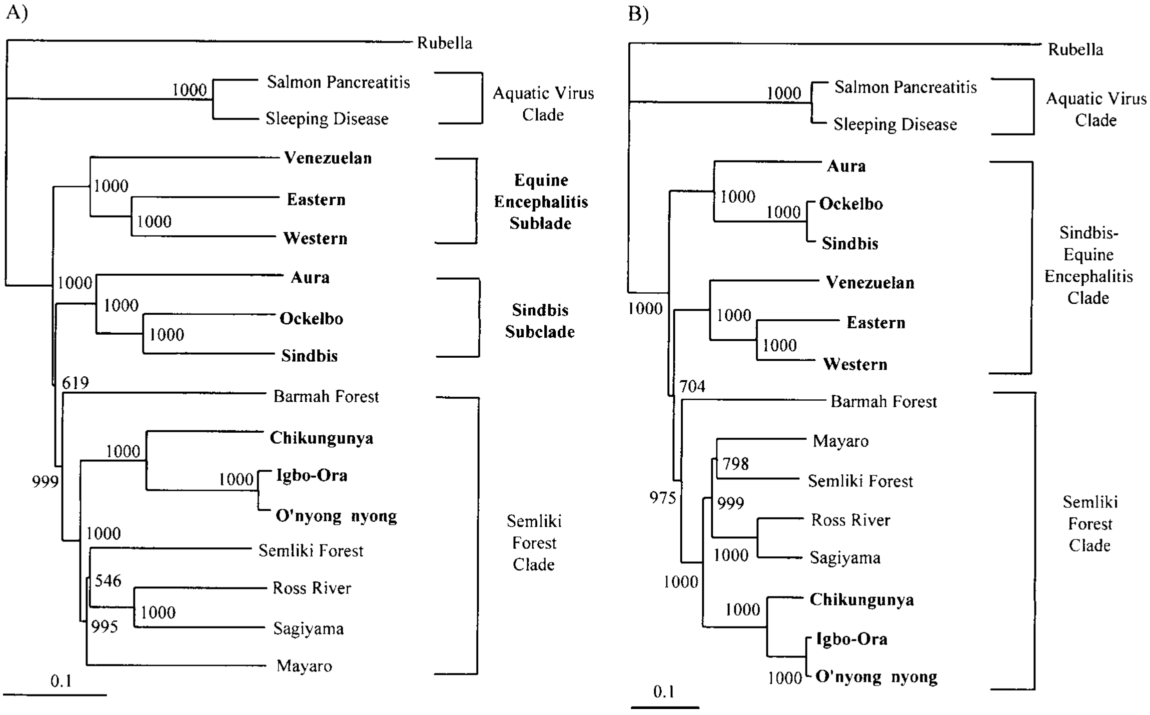

**Figure 4.** Phylograms of alphaviral non-structural polyproteins. The positions of the Sindbis-Equine Encephalitis Clade Viruses are noted. Igbo-Ora and O'nyong nyong viruses diverge from Chikungunya Virus. (A) cDNA sequences. The Equine Encephalitis and Sindbis Subclades are identified in bold. (B) Amino acid sequences physiological evidence concurs with the phylogenomic results.

The pairwise alignment of the complete genomic sequences concurs with the neighbour-joining analyses. We replicated an E1 alignment by Weaver *et al.* (1997). The results produced by MatGAT are comparable to those generated by Weaver *et al.* (1997) using PAUP (data not shown). The identity values produced by MatGAT are within 1–2% of those produced by PAUP (Hahn *et al.*, 1988; Levinson *et al.*, 1990; Weaver *et al.*, 1993; Weaver *et al.*, 1994; Weaver *et al.*, 1997; Powers *et al.*, 2001), thereby rendering our comparisons to their phylogenetic work informative.

Rubella virus, a member of the Rubivirus genus and the only other member of Family *Togaviridae,* acts as a sufficient outgroup for each of the complete genomic phylograms, as well as for the structural and non-structural phylograms (Figs. 1–4). The bootstrap values indicate a high degree of statistical significance in the relationships between the species.

Our assertions are strengthened by the results of the GenDistance analysis of the complete genomic cDNA. This program was designed not to align sequences, but to recognize patterns within alphabetical data strings. Our GenDistance cladogram shows the same three Alphavirus clades and identifies the presence of subclades within the SEE Clade. Once again, Rubella virus appears as an appropriate outgroup for rooting the phylogram.

Additionally, We have performed parsimony analysis (Felsenstein, 1989) with the complete genomic cDNA sequence. Our results (data not shown) mirror the relationships previously demonstrated by neighbour-joining analyses.

The anomalous Southern Elephant Seal Virus appeared to associate with the Equine Encephalitis Subclade in the cDNA phylogram (separating EEEV from VEEV), and the SF Clade in the amino acid phylogram. However, pairwise alignment data for every Alphavirus tested against SESV were in the average ranges of $23 \pm 3\%$ for the nucleotide sequence and $26 \pm 5\%$ for the amino acid sequence (on-line supplementary materials, Figure C). These results suggest significant genetic distance between SESV and the Equine Encephalitis Subclade. Because of these conflicting

Copyright © 2005 John Wiley & Sons, Ltd. 

**Table 2.** Proposed reclassification based on phylogenomics

| Classical alphaviral complexes | Proposed alphaviral phylogenomic clade |
|---|---|
| Barmah Forest complex | Semliki Forest clade |
| Barmah Forest Virus | |
| Eastern Equine Encephalitis complex | Sindbis–Equine Encephalitis clade |
| Eastern Equine Encephalitis Virus | Equine Encephalitis subclade |
| Middelburg Virus complex | |
| Middelburg Virus | Semliki Forest clade |
| Semliki Forest complex | Semliki Forest clade |
| Chikungunya Virus | |
| Mayaro Virus | |
| O'Nyong Nyong Virus | |
| Subtype: Igbo-Ora Virus | |
| Ross River Virus | |
| Subtype: Sagiyama Virus | |
| Semliki Forest Virus | |
| Venezuelan Equine Encephalitis complex | Sindbis–Equine Encephalitis clade |
| Venezuelan Equine Encephalitis Virus | Equine Encephalitis subclade |
| Western Equine Encephalitis complex | Sindbis–Equine Encephalitis clade |
| Aura Virus | Sindbis subclade |
| Sindbis Virus | |
| Sindbis (Babanki Virus) | |
| Sindbis (Kyzylagach Virus) | |
| Sindbis (Ockelbo Virus) | |
| Whataroa | |
| Recombinants | Sindbis–Equine Encephalitis clade |
| Buggy Creek Virus | Recombinant subclade |
| Fort Morgan Virus | |
| Highlands J Virus | |
| Western Equine Encephalitis Virus | |
| 'Unclassified' alphaviruses | Aquatic Virus clade |
| Salmon Pancreatic Disease Virus | |
| Sleeping Disease Virus | |
| Southern Elephant Seal Virus | Still unclassified |

data, the procedure was repeated with the addition of EEEV 'South America' strain, in order to enhance the relative weight of the 'North America' strain (Figure 2).

Moreover, we developed a complete structural polyprotein phylogram to further elucidate the relative distance of Barmah Forest and Southern Elephant Seal Viruses from the other members of the Semliki Forest Clade (Figure 3). We also included Middelburg Virus in this analysis. Through limited E1 partial protein phylogenetic analysis, MIDV has been shown to be a phylogenetically close relative to Semliki Forest Virus (Powers *et al.*, 2001). However, it has also been demonstrated to be antigenically different from this group (Calisher and Karabatsos, 1988). Our structural polyprotein phylogram shows high bootstrap values for all nodes and a branching pattern indicating that, while distant, BFV and MIDV are members of the SF Clade (Figure 2).

The reticulate speciation that produced the recombinant members of the SEE Clade also requires separate structural, as well as non-structural, polyprotein analysis for informative phylogenomic review. Based upon previously published works, the Western Equine Encephalitis Complex includes Aura, Sindbis, Ockelbo, WEEV, Highlands J, Fort Morgan, and Buggy Creek Viruses (Hahn *et al.*, 1988; Levinson *et al.*, 1990; Weaver *et al.*, 1997; Powers *et al.*, 2001). Our complete structural and non-structural polyprotein cDNA and amino acid phylogenomic analyses support the conclusions of the previously published literature.

Our structural cDNA and amino acid phylograms place FMV, BCV, HJ, and WEEV into their own subclade, distinct from both the Venezuelan, Eastern Equine Encephalitis, and Sindbis-like Viruses (Figure 2). The EEEV origin of the non-structural portion of the WEEV genome is indicated by its classification of WEEV into a subclade with VEEV and EEEV in the multiple pairwise alignment. These assertions are validated by the consistent bootstrap values of 1000 for the divergence of the Sindbis, Recombinant, and Equine Encephalitis subclades in both the structural and non-structural phylograms (Figs. 2 and 4).

There are two alphaviral evolutionary scenarios presently considered most parsimonious (Powers *et al.*, 2001). The 'New World Origin' hypothesis consists of three movements between the Old and New World: 1) an ancestor of the SF Clade was relocated from the New to the Old World; 2) an ancestor of SIN was relocated from the Old to the New World; 3) an ancestor of MAYV was relocated from the Old to the New World. The 'Old World Origin' hypothesis disagrees only with the first point of the New World Origin hypothesis; relocations two and three remain the same. The Old World Origin hypothesis posits that an ancestor of the Equine Encephalitis Viruses relocated from the Old to the New World.

Copyright © 2005 John Wiley & Sons, Ltd.

Mayaro and Aura Viruses (both geographically New World viruses) appear within Old World clades or subclades in each of our phylograms; therefore, we concur with events two and three of the hypotheses. Though the work of Powers *et al.* (2001) addresses Salmon Pancreatic Disease, Sleeping Disease, and Southern Elephant Seal Viruses, it makes little attempt to describe these species within either of these hypotheses.

In light of our conclusions, we have revised the theoretical cladogram (Weaver *et al.*, 1997) of alphaviral evolution to include SPDV, SDV, and SESV, showing divergence of these viruses prior to the events forming the Semliki Forest and Sindbis-Equine Encephalitis Clades (Figure 5). Based upon our complete genomic sequence analyses, we have modified the phylogenetic relationships in the Semliki Forest Complex to add Mayaro and Barmah Forest Viruses and show divergence of Chikungunya Virus, to form O'nyong nyong and Igbo-Ora Viruses (Figure 5). Lastly, we have removed subtypes of the major viruses from our cladogram to clarify our phylogenomic relationships.

Our three-clade taxonomical designation takes the methodology of alphaviral phylogenetic study in the direction of phylogenomics. Complete genomic, structural polyprotein, and non-structural polyprotein sequence analyses are able to elucidate alphaviral phylogenomics on a broad scale. These investigations provide a basis for more detailed substrain explorations, constrained only by availability of sequence data. As more Alphavirus sequencing projects are completed, and more alphaviruses are isolated, a more conclusive picture of alphaviral phylogeny may be drawn.

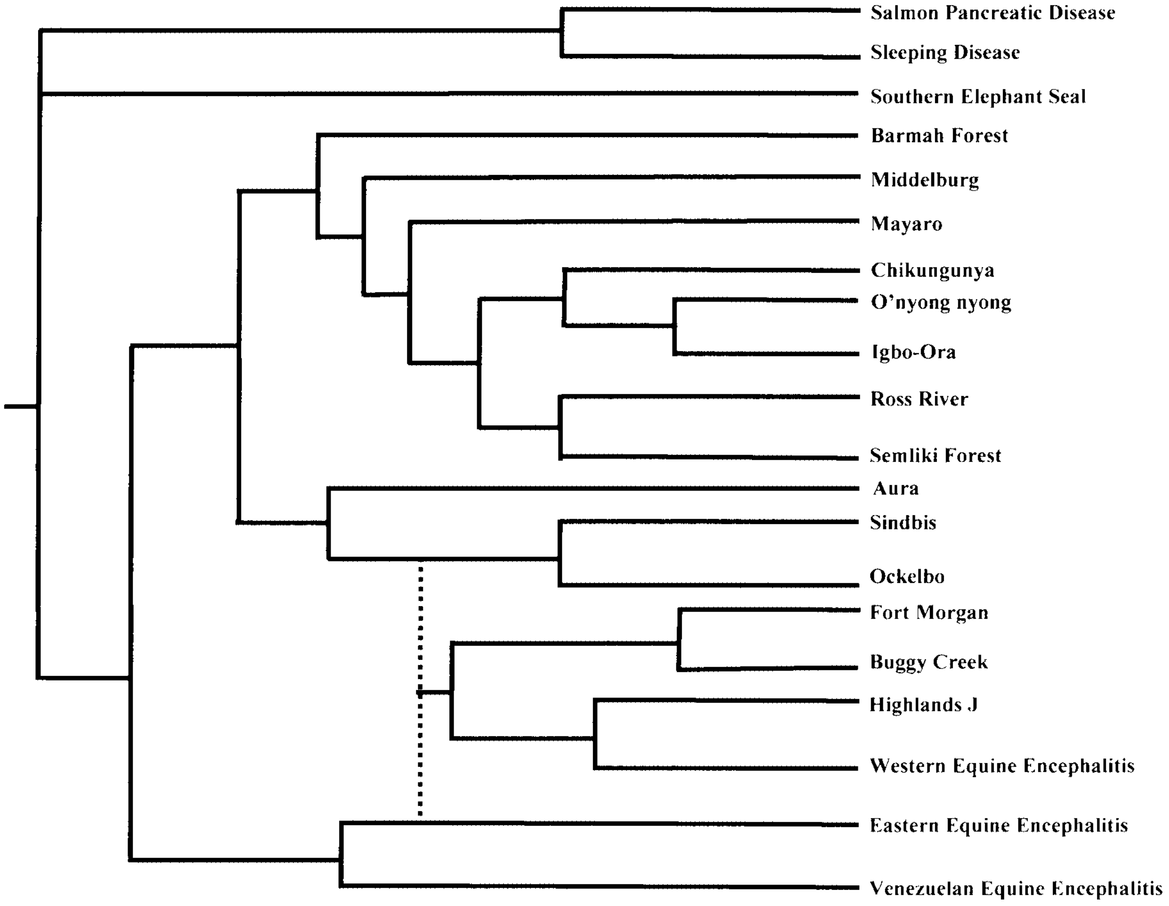

**Figure 5.** Proposed model cladogram of hypothetical alphaviral evolution. This tree was generated by hand and is based upon the phylogenomic analyses of this present study. The dashed line represents putative recombination between Eastern Equine Encephalitis and Sindbis Virus progenitors

Copyright © 2005 John Wiley & Sons, Ltd. 

### Acknowledgements

The Authors wish to thank Dr Scott Kight for his thoughtful suggestions on this manuscript. We also wish to thank Lisa Campanella for her generous help in editing this article. This research was supported by a graduate assistantship from Montclair State University.

### References

Boucher P, Castric J, Laurencin F. 1994. Observation of virus-like particles in rainbow-trout *Onchornychus mykiss* infected with sleeping disease virulent material. *Bull Eur Assoc Fish Pathol* **14**: 215–216.

Boucher P, Laurencin F. 1996. Sleeping disease and pancreas disease: comparative histopathology and acquired cross-protection. *J Fish Dis* **19**: 303–310.

Calisher C, Monath T, Muth D, *et al*. 1980. Characterization of Fort Morgan Virus, an alphavirus of the Western Equine Encephalitis Virus Complex in an unusual ecosystem. *Am J Trop Med Hyg* **29**: 1428–1440.

Calisher C, Karabatsos N. 1988. *Arbovirus Serogroups: Definition and Geographic Distribution.* CRC Press: Boca Raton, FL.

Calisher CH, Karabatsos N, Lazuick J, Monath T, Wolff K. 1988. Reevaluation of the Western Equine Encephalitis antigenic complex of alphaviruses (Family *Togaviridae*) as determined by neutralization tests. *Am J Trop Med Hyg* **38**: 447–452.

Campanella J, Bitincka L, Smalley J. 2003. MatGAT: an application that generates similarity/identity matrices using protein or DNA sequences. *BMC Bioinformat* **4**: 29.

Chen X, Kwong S, Li M. 2000. A compression algorithm for DNA sequences based on approximate matching. Proceedings of the Fourth Annual International Conference on Computational Molecular Biology (RECOMB), Tokyo, Japan; 107–118.

Christie K, Fryand K, Holtet L, Rowley H. 1998. Isolation of pancreas disease virus from farmed Atlantic salmon, *Salmo salar* L., in Norway. *J Fish Dis* **21**: 391–394.

Felsenstein J. 1985. Confidence limits on phylogenies: an approach using the bootstrap. *Evolution* **39**: 783–791.

Felsenstein J. 1989. PHYLIP phylogeny inference package. *Cladistics* **5**: 164–166.

Frey TK. 1994. Molecular biology of rubella virus. *Adv Virus Res* **44**: 69–160.

Frolov I, Hoffman T, Pragai B, *et al*. 1996. Alphavirus-based expression vectors: strategies and applications. *Proc Natl Acad Sci USA* **93**: 11 371–11 377.

Hahn C, Strauss E, Strauss J. 1988. Western equine encephalitis virus is a recombinant virus. *Proc Natl Acad Sci USA* **85**: 5997–6001.

Levinson R, Strauss J, Strauss E. 1990. Determination of the complete nucleotide sequence of the genomic RNA of O'Nyong Nyong Virus and its use in the construction of phylogenetic trees. *Virology* **175**: 110–123.

National Center for Biotechnology Information. 2004. **www.ncbi.nlm.nih.gov**

National Center for Biotechnology Information (BLAST). 2004. **www.ncbi.nlm.nih.gov/blast/bl2seq/bl2.html**

Page RD. 1996. TREEVIEW: an application to display phylogenetic trees on personal computers. *Comput Appl Biosci* **12**: 357–358.

Powers A, Brault A, Shirako Y, *et al*. 2001. Evolutionary relationships and systematics of alphaviruses. *J Virol* **75**: 10 118–10 131.

Saitou N, Nei M. 1987. The neighbour-joining method: a new method for reconstructing phylogenetic trees. *Mol Biol Evol* **4**: 406–425.

Schlesinger S, Dubensky T. 1999. Alphavirus vectors for gene expression and vaccines. *Curr Opin Biotechnol* **10**: 434–439.

Strauss J, Strauss E. 1994. The alphaviruses: gene expression, replication, and evolution. *Microbiol Rev* **58**: 491–562.

Strauss J, Strauss E. 1997. Recombination in alphaviruses. *Semin Virol* **8**: 85–94.

Thompson JD, Gibson TJ, Plewniak F, *et al*. 1997. The Clustal X windows interface: flexible strategies for multiple sequence alignment aided by quality analysis tools. *Nucleic Acids Res* **24**: 4872–4882.

Villoing S, Béarzotti M, Chilmonczyk S, Castric J, Brémont B. 2000. Rainbow Trout Sleeping Disease Virus is an atypical alphavirus. *J Virol* **74**: 173–183.

Weaver S, Hagenbaugh A, Bellew L, *et al*. 1993. A comparison of the nucleotide sequences of Eastern and Western Equine Encephalomyelitis Viruses with those of other alphaviruses and related RNA viruses. *Virology* **197**: 375–390.

Weaver S, Hagenbaugh A, Bellew L, *et al*. 1994. Evolution of alphaviruses in the Eastern Equine Encephalomyelitis complex. *J Virol* **68**: 158–169.

Weaver S, Wenli K, Shirako Y, *et al*. 1997. Recombinational history and molecular evolution of Western Equine Encephalomyelitis complex alphaviruses. *J Virol* **71**: 613–623.

Weston J, Villoing S, Brémont M, *et al*. 2002. Comparison of two aquatic alphaviruses, Salmon Pancreas Disease Virus and Sleeping Disease Virus, by using genome sequence analysis, monoclonal reactivity, and cross-infection. *J Virol* **76**: 6155–6163.

Copyright © 2005 John Wiley & Sons, Ltd. 
